# Characterization of *Weissella koreensis* SK Isolated from Kimchi Fermented at Low Temperature (around 0 °C) Based on Complete Genome Sequence and Corresponding Phenotype

**DOI:** 10.3390/microorganisms8081147

**Published:** 2020-07-29

**Authors:** So Yeong Mun, Hae Choon Chang

**Affiliations:** Department of Food and Nutrition, Kimchi Research Center, Chosun University, 309 Pilmun-daero, Dong-gu, Gwangju 61452, Korea; ohno1757@gmail.com

**Keywords:** *Weissella koreensis*, low-temperature, fermentation, kimchi, ADI pathway

## Abstract

This study identified lactic acid bacteria (LAB) that play a major role in kimchi fermented at low temperature, and investigated the safety and functionality of the LAB via biologic and genomic analyses for its potential use as a starter culture or probiotic. Fifty LAB were isolated from 45 kimchi samples fermented at −1.5~0 °C for 2~3 months. *Weissella koreensis* strains were determined as the dominant LAB in all kimchi samples. One strain, *W. koreensis* SK, was selected and its phenotypic and genomic features characterized. The complete genome of *W. koreensis* SK contains one circular chromosome and plasmid. *W. koreensis* SK grew well under mesophilic and psychrophilic conditions. *W. koreensis* SK was found to ferment several carbohydrates and utilize an alternative carbon source, the amino acid arginine, to obtain energy. Supplementation with arginine improved cell growth and resulted in high production of ornithine. The arginine deiminase pathway of *W. koreensis* SK was encoded in a cluster of four genes (*arcA*-*arcB*-*arcD*-*arcC*). No virulence traits were identified in the genomic and phenotypic analyses. The results indicate that *W. koreensis* SK may be a promising starter culture for fermented vegetables or fruits at low temperature as well as a probiotic candidate.

## 1. Introduction

Members of the *Weissella* genus belonging to the Leuconostocacea family are gram-positive, non-spore forming, heterofermentative, and short-rod bacteria [1]. *Weissella* can be isolated from a variety of sources, including fermented foods, milk, vegetable, soil, and the gastrointestinal tracts of humans and animals [2]. To date, the *Weissella* genus comprises 19 validly described species [2]. *Weissella koreensis* isolated from kimchi, a traditional Korean fermented food, was first proposed as a novel species belonging to the *Weissella* genus in 2002 by Lee et al. [1].

Several health benefits or functions related with *Weissella* species, including anti-tumor, anti-obesity, and immuno-modulatory effects, have been reported [3,4,5]. Although human infections caused by *Weissella* species are rarely reported, some *Weissella* species were reported to be involved in disease outbreaks, including endocarditis, sepsis, and fish mortality [6,7]. Thus, any strain intended to be used as a starter culture or probiotic should be properly evaluated in terms of safety.

Members of the *Weissella* genus have been reported as one of the major lactic acid bacteria (LAB) responsible for kimchi fermentation [8]. Among *Weissella* species, *Weissella confusa* and *Weissella cibaria* species are being extensively studied for their use as probiotics based on functional and genomic characteristics [2,9], whereas only few studies have been conducted on the functional, genomic, and metabolic features of *W. koreensis* [4,10].

The aim of this study was to identify a dominant LAB species in kimchi fermented at 1ow temperature as well as investigate its safety and functionality via biologic and genomic analyses for its potential use as a starter culture or probiotic. For this, we isolated a total of 50 LAB strains as dominant LAB from various kimchi samples, which were fermented at −1.5~0 °C for 2~3 months. One strain was selected among the isolates, after which its whole genome was sequenced, and genomic features analyzed. Furthermore, its phenotypic characteristics such as carbohydrate assimilation, growth at different temperatures, acid-alkali tolerance, amino acid (arginine) catabolism, and virulence traits were investigated and further compared with its genome analysis data.

## 2. Materials and Methods 

### 2.1. Isolation and Identification of LAB

Freshly made kimchi was collected from different locations in South Korea and fermented at −1.5~0 °C (Kimchi refrigerator, LG, Changwon, Korea) for 2~3 months. The resulting kimchi sample was macerated, and the kimchi slurry was filtered through a sterile thin cloth [11]. The kimchi filtrate was then serially diluted with sterile-distilled water and spread onto de Man, Rogosa, and Sharpe (MRS; Difco, Sparks, MD, USA) +2% CaCO_3_ agar. The plates were incubated at 30 °C for 2~3 days, after which colonies forming a clear zone on the MRS+2% CaCO_3_ agar were tentatively selected as a LAB strain. The isolates were identified by gram-staining, catalase test, morphological observation under a microscope (Eclipse 55i, Nikon, Tokyo, Japan), and determination of 16S rRNA gene sequences [12,13]. The determined 16S rRNA gene sequences were compared with sequences available in the GenBank database (https://www.ncbi.nlm.nih.gov/) using CLUSTAL W (https://www.genome.jp/tools-bin/clustalw).

### 2.2. Polymerase Chain Reaction (PCR)-Denaturing Gradient Gel Electrophoresis (DGGE)

The prepared kimchi filtrate was centrifuged (13,475× *g*, 5 min, 4 °C), the pellet washed, and DNA extracted using a genomic DNA preparation kit (Qiagen, Hilden, Germany). The extracted DNA was then used as a template for PCR amplification of the 16S rRNA gene using 27F and 1492R 16S universal primers [14]. The purified amplicon was used as a template for nested PCR targeting the V3 region of the 16S rRNA gene using the DGGE primers of *Lac1* and GC *Lac2* [15]. DGGE was conducted using 8% (*w*/*v*) acrylamide with the Dcode system (BioRad, Hercules, CA, USA). Bands of interest were extracted from gels, reamplified using the DGGE primers of *Lac1* and GC *Lac 2*, and sequenced using an automated DNA sequencer (Applied Biosystem, Forster City, CA, USA). The determined partial ribosomal DNA sequences were queried by Blast searches of the GenBank database to identify the closest known relatives. 

### 2.3. Whole Genome Sequencing, Assembly, and Annotation

Whole genomic DNA from the LAB isolate was extracted using DNeasy Blood & Tissue Kits (Qiagen). The extracted DNA was completely sequenced by Macrogen (Seoul, Korea) using PacBio RS single-molecule real-time (SMRT) sequencing and Illumina HiseqXten sequencing. The PacBio SMRT reads were assembled using the hierarchical genome assembly process, and errors in PacBio sequencing were corrected by paired-end reads obtained by Illumina sequencing. The complete genome of the LAB isolate consisting of a chromosome and plasmid were deposited in GenBank under the accession numbers CP043431-2. Sequences were further annotated by RAST server using the classic scheme (https://rast.theseed.org/FIG/rast.cgi). Protein coding sequences (CDSs) encoded on the plasmid were annotated by using NCBI BlastX (https://blast.ncbi.nlm.nih.gov/Blast.cgi).

### 2.4. Plasmid Preparation and Digestion

Plasmid in the LAB isolate was extracted by using a QIAprep spin miniprep kit (Qiagen). The isolated plasmid DNA was digested with restriction enzymes, *Bgl* II and *Blp* I, at 37 °C for 1 h. The plasmid digestion mixtures were separated by electrophoresis on 1% (*w*/*v*) agarose gel stained with ethidium bromide.

### 2.5. Phenotypic Properties

#### 2.5.1. Carbohydrate Assimilation

Carbohydrate assimilation of the LAB isolate was investigated using an API 50 CHL (BioMérieux, Marcy l’Etoile, France) according to the manufacturer’s instructions. *W. koreensis* KACC 11853^T^, obtained from the Korean Agricultural Culture Collection (Wanju, Korea), was used as a control.

#### 2.5.2. Growth at Different Temperatures

Growth of the LAB isolate at different temperatures was observed in MRS broth at −1.5, 5, 10, 15, 25, and 30 °C for 24~2016 h. Growth was determined by monitoring the absorbance at 600 nm (A_600_; Ultrospec 2100 pro, Biochrom, Cambridge, UK). Growth of viable cells after cultivation at different temperatures was also measured; briefly, the culture was serially diluted, spread on MRS (Difco) agar, and incubated at 30 °C, after which the colonies on the plate were counted.

#### 2.5.3. Cell Viability According to pH Level

To determine the acid and alkali tolerances of the LAB isolate, the isolate was adjusted to pHs ranging from 3.0~10.0 for 24~48 h. Specifically, the isolate was cultivated in 5 mL of MRS broth for 24 h, centrifuged (9950× *g*, 5 min, 4 °C), and then resuspended in 5 mL of MRS broth adjusted to pHs of 3.0, 4.0, 5.0, 8.0, 9.0, and 10.0. MRS broth without pH adjustment (pH 6.5) was used as a control. Subsequently, the suspensions were incubated at 30 °C for 24 or 48 h, after which cell viabilities of suspensions were determined. Homofermentative LAB, *Lactobacillus plantarum* AF1 (GenBank accession No. FJ386491), which shows strong acid tolerance [16], was used as a control LAB. 

#### 2.5.4. Arginine Catabolic Ability

The LAB isolate was cultivated in MRS, MRS+1% arginine, and MRS+1% citrulline broths (pH 6.5) at 30 °C for 12, 18, 24, 48, and 72 h. Thereafter, viable cells, total cells (A_600_), pH level, and acidity of the culture were measured. Simultaneously, organic acids, ethyl alcohol, amino acids (arginine, citrulline, and ornithine), and ammonia contents of the culture supernatant were measured. 

Organic acids and amino acids were analyzed by HPLC (Ultimate 3000, Thermo Scientific Dionex, Sunnyvale, CA, USA). For organic acid and ethyl alcohol analysis, an Aminex 87H column (300 × 10 mm; Bio-Rad) was used, and the elution solvent was 0.01 N H_2_SO_4_ at a flow rate of 0.5 mL/min. Organic acids and ethyl alcohol were detected using an RI detector (RefractoMAX520, Tokyo, Japan) [17]. For amino acid analysis, the filter-sterilized culture broth was derivatized using O-phthalaldehyde (OPA)-fluorenylmethyl chloroformate (FMOC) [18] and then analyzed using the HPLC system (Ultimate 3000) equipped with a UV detector and FL detector (Agilent 1260, Agilent, Santa Clara, CA, USA). An Inno C_18_ column (5 μm, 4.6 × 150 mm; Youngjin, Seongnam, Korea) was used. The mobile phase was prepared with A solution (40 mM sodium phosphate (pH 7.0)) and B solution (water:acetonitrile:methanol = 10:45:45). The program was initiated using solutions A:B = 95:5 (*v*/*v*) for 0~24 min, 45:55 for 24~25 min, 10:90 for 25~34.5 min, and 95:5 after 34.5 min at a flow rate of 1.5 mL/min [17]. Ammonia was analyzed by cation-chromatography (Dionex ICS 5000, Thermo Scientific Dionex) equipped with a suppressed conductivity detector and cation self-regenerating suppressor (CSRS-Ultra, 4 mm; Thermo Scientific Dionex) in recycle mode. An Ionpac CS16A column (4 × 250 mm, Thermo Scientific Dionex) was used. Mobile phase was 40 mM methane sulfonic acid at a flow rate of 1 mL/min. 

#### 2.5.5. Biogenic Amine Production

LAB were incubated in MRS broth supplemented with a precursor amino acid (1% arginine, ornithine, or histidine) at 30 °C for 48 h, after which the culture was centrifuged (9950× *g*, 15 min, 4 °C). The supernatant was further filter-sterilized (0.4 μm pore size, Advantec, Dublin, CA, USA), freeze-dried, and then concentrated 5-fold in distilled water. The concentrated sample (2 mL) was added to 10 mL of 5% trichloroacetic acid-0.4 M perchloric acid, after which 0.2 mg of 1,7-diaminoheptane was added as an internal standard. The mixture was homogenized for 5 min, derivatized using benzoyl chloride, dissolved in 0.5 mL of 50% methanol, and then injected (20 μL) for HPLC analysis [19]. The HPLC system (Waters 2695, Waters, Boston, MA, USA) consisted of Waters 2996 photodiode array detector (254 nm) along with Waters Empower software. A SunFire C_18_ column (3.5 μm, 4.6 × 150 mm; Waters) was used at a flow rate of 0.4 mL/min. The program was initiated with 50% methanol, maintained at 50% methanol for 15 min, increased to 90% methanol for 10 min, decreased to 50% methanol for 5 min, and then maintained at 50% methanol for an additional 5 min [20].

### 2.6. Statistical Analysis

Data are presented as the means and standard deviations (means ± SD) of two independent experiments performed in duplicate. Statistical analyses on the data were performed using the Duncan’s multiple range test of SPSS version 26.0 for Windows (SPSS, Chicago, IL, USA) with the significance determined at *p* < 0.05.

## 3. Results and Discussion

### 3.1. Kimchi Samples and Isolation of LAB

Mesophilic LAB play a key role in lactic acid fermentation of dairy foods, which occurs at 37~42 °C [21]. On the other hand, kimchi is generally fermented at low temperature (−1~10 °C), although sometimes it is fermented at room temperature (15~20 °C) to accelerate the fermentation process [8,11]. In Korea, to ensure and maintain quality, a special kimchi refrigerator equipped with a fermentation mode at 6~7 °C and storage mode at −2~−1 °C is used for the long-term storage of kimchi [22].

Analyses have shown that LAB communities belonging to the genera *Leuconostoc*, *Lactobacillus*, and *Weissella* are mainly responsible for kimchi fermentation [23]. However, the dominant LAB strain in fermented kimchi can vary depending on the fermentation temperature, as LAB present in kimchi materials have different growth properties according to temperature [8,22]. In this study, we tried to identify and characterize a key LAB in kimchi fermented at low temperature (around 0 °C) for long-term storage. Thus, a total of 45 freshly made kimchi samples (pH 5.81~6.39) were collected from 38 different regions in Korea and then immediately fermented at −1.5~0 °C for 2~3 months (Supplementary Table S1). After fermentation, pHs of the kimchi samples ranged between 4.18~4.39 (data not shown), which indicates ripened kimchi.

Isolation of LAB from the kimchi samples was performed next. Relatively tiny-shaped colonies with a faint clear zone on MRS+2% CaCO_3_ plate were detected as dominant LAB (accounting for 38~96% of the plates) from all 45 kimchi samples, whereas the others showed definitely larger colonies with a distinct clear zone (data not shown). A total of 50 LAB strains were isolated from 45 kimchi samples (Supplementary Table S1). All isolates were gram-positive, catalase-negative, and short rod-shaped cells. When 16S rRNA gene sequences (1,268~1,523 bp) of the 50 isolates were compared with those of LAB type strains in GenBank, all 50 sequences showed 100% homology with those of *W. koreensis* JCM11263^T^ (Supplementary Table S1). This result is very unique, considering the 16S rRNA gene sequences of other LAB species such as *Leuconostoc* and *Lactobacillus* species show high similarities (greater than 99.54% but not 100%) with those of type strains in GenBank [24,25]. The reason that all 50 16S rRNA gene sequences of the isolates showed 100% homology with each other along with type strain in GenBank should be investigated via further experimentation.

**Figure 1:**
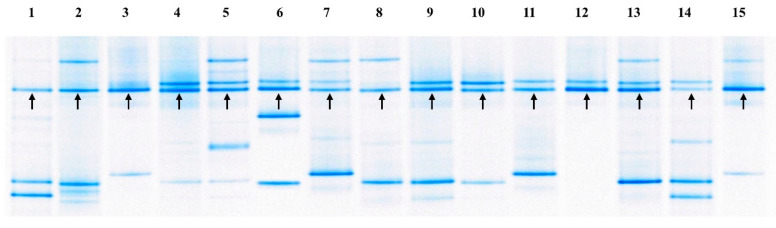
PCR-DGGE patterns of 16S V3 rRNA gene sequences in the kimchi samples. 1: Kimchi 1, 2: Kimchi 3, 3: Kimchi 5, 4: Kimchi 10, 5: Kimchi 13, 6: Kimchi 17, 7: Kimchi 21, 8: Kimchi 23, 9: Kimchi 27, 10: Kimchi 30, 11: Kimchi 33, 12: Kimchi 37, 13: Kimchi 39, 14: Kimchi 41, 15: Kimchi 43. The closest relative of the fragments (arrow-indicated) was determined and compared using sequences from GenBank as described in the Materials and Methods.

Among the 50 isolates, we selected 15 isolates from 45 kimchi samples according to sampling location; LAB 1, 3, and 6 from Seoul/Gyeonggi, LAB 11, 14, and 18 from Gangwon, LAB 23, 26, and 31 from Chungcheong, LAB 34 and 37 from Busan/Gyeongsang, and LAB 41, 43, 45, and 48 from Gwangju/Jeolla (Supplementary Table S1). Subsequently, to confirm that the selected 15 isolates are indeed dominant LAB in each kimchi sample, analysis of bacterial communities in the kimchi samples was performed by PCR-DGGE (Figure 1). Microbial population profiles of the kimchi samples were different from each other. However, the arrow-indicated bands identified (with 100% identity) as *W. koreensis* were detected as a ticker band from all 15 kimchi samples (Figure 1). The results demonstrate that *W. koreensis* was a dominant LAB in kimchi fermented at low temperature (−1.5~0 °C for 2~3 months). Kim also reported that *W. koreensis* is easily detected in kimchi stored at −1 °C for 1~2 months [26].

Finally, one isolate (LAB 6) designated as *W. koreensis* SK among the 15 isolates was selected based on carbohydrate assimilation (Supplementary Table S2) for further experimentation.

### 3.2. Genome Properties of W. koreensis SK

**Figure 2:**
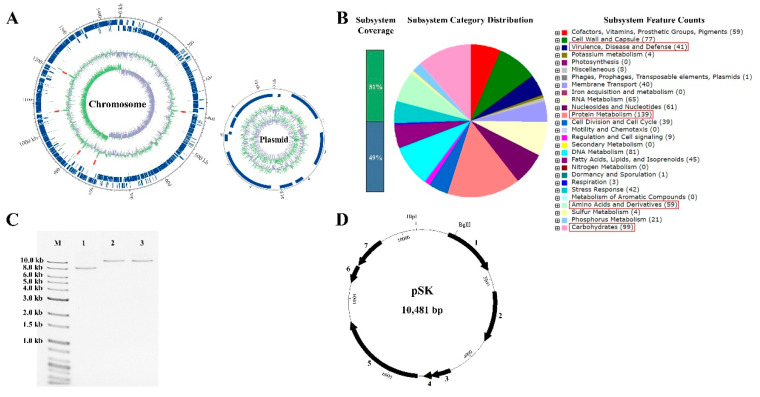
Genomic features of *W. koreensis* SK. (**A**) Circular representation of the chromosome and plasmid genome of *W. koreensis* SK. Marked characteristics are shown from outside to the center; CDS on forward strand, CDS on reverse strand, tRNA, rRNA, GC content and GC skew. (**B**) Subsystem feature of the genomic sequence of *W. koreensis* SK analyzed with RAST server. Out of 1334 coding sequences predicted by RAST server, the subsystem coverage is 51% (the green bar), which contributes to a total of 237 subsystems. The blue bar refers to the % of proteins that are not included in the subsystems. (**C**) Agarose gel electrophoresis of pSK. M, size marker; 1, ccc pSK; 2, linear pSK digested with *Bgl* II, 3; linear pSK digested with *Blp* I. (**D**) Physical and genetic map of pSK from *W. koreensis* SK. Orientation of deduced CDSs are marked by black arrows.

A total of four complete genome sequences of *W. koreensis* (SK in this study, KACC 15510, WiKim0080 and CBA3615) can be obtained from GenBank as of now. The genomes of the four strains were compared and their general features are summarized (Table 1). The complete genome of *W. koreensis* SK was found to consist of a circular single chromosome (1,451,607 bp) and one circular plasmid (10,481 bp) with 35.5% and 36.9% G+C contents, respectively (Figure 2A, Table 1). A total of 1318 protein coding genes were identified among the 1389 predicted total genes, along with 71 RNA genes on the chromosome and 11 coding genes on the plasmid. The general features of the four strains are slightly different from each other; WiKim0080 strain harbors the biggest genome (1,537,013 bp) along with two plasmids, while KACC 15510 harbors the smallest genome (1,441,470 bp).

All protein coding sequences (CDSs) of *W. koreensis* SK were functionally annotated by subsystems based on RAST server (Figure 2B). The function annotated results indicate that genes in the *W. koreensis* SK chromosome were related with protein metabolism (15.57%), carbohydrates (10.97%), DNA metabolism (9.07%), cell wall and capsule (8.82%), RNA metabolism (7.28%), nucleosides and nucleotides (6.83%), amino acids and derivatives (6.61%), etc. Genes for protein metabolism, which account for the highest percentage of the SK strain genome, were shown to be mainly related with protein biosynthesis to maintain cell metabolism. Regarding RAST functional annotation for carbohydrates, *W. koreensis* SK was shown to harbor genes for glucose metabolism as a central carbohydrate, followed by chitin/N-acetylglucosamine, lactose/galactose, glycerate, lactate, glycerol/glycerol-3-phosphate, mannose, ribose, xylose, gluconate/ketogluconate, deoxyribose/deoxynucleoside, and arabinose. Regarding functional annotation for amino acids and derivatives, *W. koreensis* SK was found to harbor genes related with arginine degradation (37.29%), polyamine metabolism (22.03%), methionine biosynthesis and degradation (22.03%), as well as glutamine, glutamate, and asparagine biosynthesis (8.47%).

For the safety assessment of *W. koreensis* SK, genes related with biogenic amine production, hemolysis, and disease and defense such as antibiotic resistance were investigated. RAST analysis identified a gene encoding a hemolysin Ⅲ in *W. koreensis* SK. There are multiple reports that most *Weissella* species are vancomycin-resistant, as they harbor a D-alanine-D-alanine ligase gene [9]. The D-alanine-D-alanine ligase gene contributing to vancomycin resistance was also identified in *W. koreensis* SK. However, the analysis did not detect any other genes related with biogenic amine production, toxins, production of superantigens, antibiotic resistance except for vancomycin, or disease and defense in the genome of *W. koreensis* SK (Figure 2B).

On the other hand, restriction enzyme sites on plasmid pSK were investigated using NEB Cutter V2.0 tools (http://nc2.neb.com/Nebcutter2/), and 31 single-cutter restriction sites were identified. pSK was extracted from *W. koreensis* SK by digestion with *Bgl* II and *Blp* I at the predicted single-cutter restriction site on pSK. One circular plasmid comprising 10,481 bp in *W. koreensis* SK was confirmed by this plasmid digestion (Figure 2C). Among the 11 coding genes on pSK, seven putative proteins (designated ORF 1 to ORF 7) showing greater than 98% homologies with the CDSs in BlastX with 100% query cover were functionally annotated as mobilization protein, RepB for replication of plasmid, copper transport repressor, heavy-metal associated domain-containing protein, cadmium-translocating P-type ATPase, glyoxalase/extradiol dioxygenase family protein, and fructose permease (Table 2).

### 3.3. Phenotypic Characteristics of W. koreensis SK

#### 3.3.1. Carbohydrate Assimilation

Regarding carbohydrate assimilation, *W. koreensis* strains were positive for 6~8 carbohydrates, which is a substantially lower number than other LAB species. For instance, *Lactobacillus* or *Leuconostoc* species can utilize numerous (15~23) carbohydrates [25,29,30]. The 15 isolates and *W. koreensis* KACC 11853^T^ as a control (Supplementary Table S2) were positive for ribose, D-glucose, D-mannose, and N-acetyl glucosamine as well as weakly positive for gluconate. Differences in carbohydrate assimilation among LAB were observed for L-arabinose, D-xylose, and D-fructose; 12 isolates showed the same carbohydrate assimilation profile as *W. koreensis* KACC 11853^T^, while LAB 3 was only negative for L-arabinose, LAB 23 was only negative for D-xylose, and strains LAB 3, 6, and 23 were negative for D-fructose. From the results of carbohydrate assimilation, we selected LAB 6, identified and designated as *W. koreensis* SK in Section 3.1, which showed the same carbohydrate assimilation profile as *W. koreensis* KACC 11853^T^ except for fructose assimilation, for further experiments. 

When the results of carbohydrate assimilation were compared with gene annotation for carbohydrates, *W. koreensis* SK was shown to ferment L-arabinose, ribose, D-xylose, D-glucose, D-mannose, N-acetylglucosamine, and gluconate along with harboring genes for utilization of those carbohydrates. *W. koreensis* SK was also found to harbor genes related with glycerol, galactose, and lactose utilization, but these genes were not phenotypically expressed. Specifically, in the case of lactose utilization, it is well known that LAB can utilize lactose, but it is more common in dairy originated LAB. As kimchi does not contain lactose, LAB in kimchi might not require lactose as a carbon source. We previously reported the lactose-negative metabolism of kimchi-originated LAB; most LAB such as *Leuconostoc* and *Weissella* species isolated from kimchi are lactose-negative [16,30]. We also previously reported that the more evolutionally developed strains might have deleted or turned off expression of lactose metabolic genes [16]. However, the current study indicates that kimchi-originated LAB, *W. koreensis* SK, still retain lactose utilization genes (eight copies) without any phenotypic gene expression. 

On the other hand, *W. koreensis* SK was determined to be fructose-negative, whereas the other 13 *W. koreensis* strains, including KACC 11853^T^, were fructose-positive (Supplementary Table S2). For LAB to metabolize fructose as a carbon source, fructose should be transported inside of the cell by the fructose transport system, after which fructose can be converted into fructose-6-phosphate by fructose kinase (*fk*) or mannitol by mannitol dehydrogenase (*mdh*) [23]. However, neither of these fructose-metabolizing genes (*fk* and *mdh*) were detected in the genome of *W. koreensis* SK, whereas one copy of fructose-permease gene (ORF 7) was identified in plasmid pSK (Table 2). In addition, most *Lactobacillus* and *Leuconostoc* species reported to be fructose-positive [23] were found to usually harbor several copies of fructose-permease genes encoded on their chromosomes (https://rast.theseed.org/ FIG/rast.cgi). The results suggest that some *W. koreensis* strains may have deleted fructose-metabolizing genes during their evolutionary process and now utilize other carbon sources instead of fructose to obtain energy. Thus, a fructose-permease gene encoded on plasmid pSK may be considered as an evolutionary trace of the past fructose-metabolizing ability of *W. koreensis* SK.

#### 3.3.2. Growth at Different Temperatures

**Figure 3:**
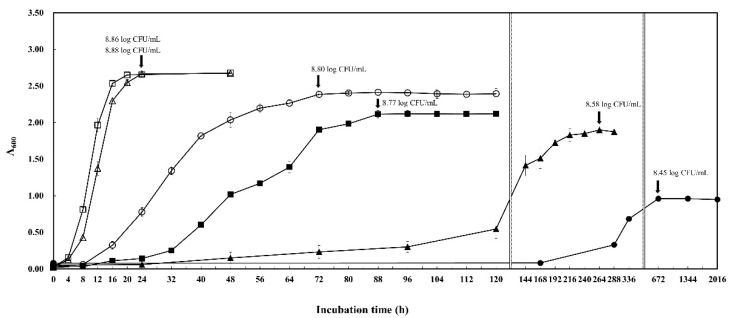
Growth of *W. koreensis* SK at different temperature. *W. koreensis* SK was incubated in MRS broth at −1 °C (●); 5 °C (▲); 10 °C (■); 15 °C (○); 25 °C (△); 30 °C (□) for 24~2016 h.

Growth of *W. koreensis* SK at different temperatures was investigated (Figure 3). Optimum growth temperature of *W. koreensis* SK was 25~30 °C, but *W. koreensis* SK grew well at 5 °C (A_600_:1.90, and 8.58 CFU/mL for 264 h of cultivation) and even at −1.5 °C (A_600_:0.96 and 8.45 log CFU/mL for 672 h). As shown in Figure 3, *W. koreensis* SK, reported as a psychrophilic bacterium [8], was able to grow well under mesophilic as well as psychrophilic conditions.

#### 3.3.3. Tolerance to Acid and Alkali Conditions

**Figure 4:**
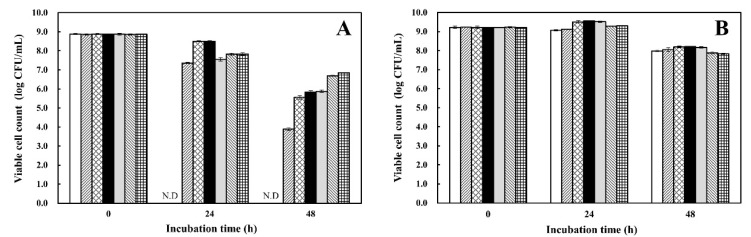
Acid and alkali tolerances of *W. koreensis*. Overnight-cultured *W. koreensis* SK (**A**) and *L. plantarum* AF1 (**B**) in MRS broth (pH 6.5) were harvested, and each cell pellet was resuspended in MRS broth adjusted to pH 3.0 (□), 4.0 (▨), 5.0 (▩), 6.5 (■, as a control), 8.0 (■), 9.0 (▧), or 10.0 (▦). The suspension was incubated at 30 °C for 24 or 48 h. Thereafter, viable cells of the suspension were determined.

As shown in Figure 4, the acid tolerance of *W. koreensis* SK was significantly weaker than that of *L. plantarum* AF1, which showed strong acid tolerance (94.3~99.6% survival rate) after treatment with phosphate-buffered saline or simulated gastric juice (pH 2.5) for 1 h [16]. Cell viability of *W. koreensis* SK was dramatically reduced between pH 3.0~4.0; no surviving *W. koreensis* SK cells were detected upon treatment at pH 3.0 for 24 h, and reduced (approximately 90-folds) cell viability was observed upon treatment at pH 4.0 for 48 h compared to the control (pH 6.5) at 48 h. On the other hand, the strong acid-tolerant LAB strain *L. plantarum* AF1 was stable within the pH range of 3.0~6.5 for 24~48 h. It has been reported that members of *Lactobacillus* and *Weissella*, including *L. sakei*, *L. plantarum*, and *W. koreensis*, show strong acid tolerance, and they become more dominant as the kimchi fermentation environment becomes more acidic [23]. However, according to the results in Figure 4, *L. plantarum* AF1 was strongly acid-tolerant while *W. koreensis* SK was not.

*L. plantarum* AF1 showed significantly higher stability in MRS for 48 h than *W. koreensis* SK. Cell viability of *L. plantarum* AF1 slightly was reduced depending on the increase in pH from 8.0 to 10.0. However, the alkali tolerance of *W. koreensis* SK was determined to be quite high, and reduction of SK viable cells at pH 9.0~10.0 was significantly less compared to that below pH 8.0.

#### 3.3.4. Biogenic Amine (BA) Production and Other Virulence Traits

Although BAs are required for many critical biological functions, excessive human consumption of BAs can have toxicological effects [31]. BAs such as agmatine, putrescine, and histamine can be produced by microbial decarboxylation of amino acids, arginine, ornithine, and histidine, respectively [31]. In this study, we examined whether or not *W. koreensis* SK produces BAs via amino acid decarboxylation or in combination with decarboxylation and the ADI pathway. As shown in Supplementary Figure S1, no BA was detected among the *W. koreensis* SK cultures, which supports the gene analysis in which *W. koreensis* SK was demonstrated as lacking biogenic amine-forming genes. However, some *Weissella* strains have been reported to produce BAs such as histamine and putrescine [32]. The selection of LAB starters lacking pathways for BA accumulation is essential to develop high-quality foods with reduced contents of these toxic compounds. Thus, it was proposed that the inability of a strain to synthesize BAs is an important selection criterion for starter cultures [32].

In our previous study, the potential virulence of *W. koreensis* SK was investigated; *W. koreensis* SK did not show α- or β-hemolysis activity [33]. However, in this study, a gene encoding hemolysin Ⅲ was identified in *W. koreensis* SK. Most LAB species have been recognized as Generally Recognized as Safe (GRAS) by the U.S. Food and Drug Administration [34] or have attained Qualified Presumption of Safety (QPS) status by the European Commission-European Food Safety Authority (EFSA) [35]. However, *Weissella* species have not yet attained QPS status [36], and thus breakpoints for antibiotics for *Weissella* species have not been made available by the EFSA. The minimal inhibitory concentrations (MICs) (0.5~8 µg/mL) [33] of antibiotics (ampicillin, gentamycin, kanamycin, streptomycin, erythromycin, tetracycline, and chloramphenicol) for *W. koreensis* SK have been reported to be significantly lower than those for other *Weissella* species, including *W. koreensis*, as determined by previous reports [37,38]. Breakpoints (MICs) of vancomycin for LAB such as *Lactobacillus* and *Leuconostoc* species are not required according to the guidelines of the EFSA [39], as most LAB are intrinsically vancomycin-resistant with MICs greater than 512 µg/mL [16,33]. Regarding vancomycin, *W. koreensis* SK showed a significantly lower MIC (128 µg/mL) [33], although our results show that it harbors a vancomycin-resistant gene. These results indicate that that *W. koreensis* SK is susceptible to all antibiotics tested except vancomycin. Based on these results, it can be concluded that *W. koreensis* SK is safe for human consumption as a starter culture or a probiotic.

#### 3.3.5. Growth of *W. koreensis* SK in MRS Supplemented with Arginine and Citrulline

**Figure 5:**
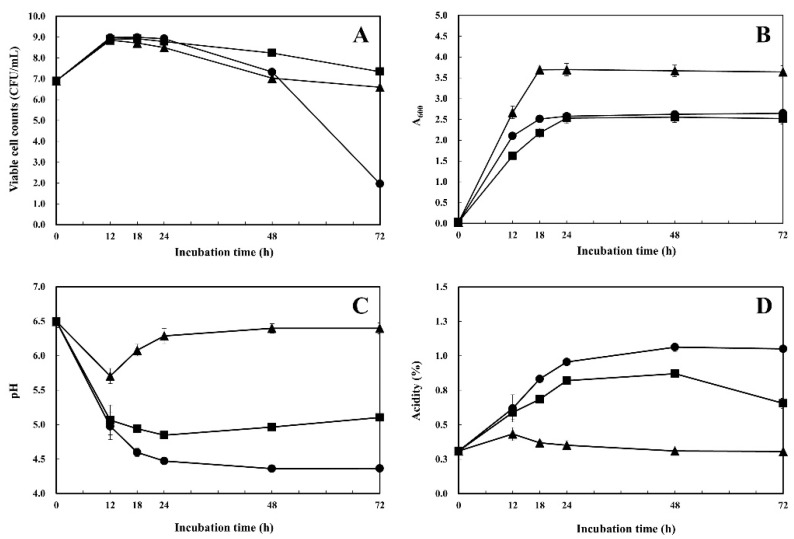
Growth, pH, and acidity changes of *W. koreensis* SK. *W. koreensis* SK was cultivated in MRS (●); MRS+1% arginine (▲); MRS+1% citrulline (■) at 30 °C for 72 h. Viable cell (**A**), total cell (**B**), pH (**C**), and acidity (**D**) were determined.

To investigate the arginine catabolic features of *W. koreensis* SK, strain SK was cultivated in MRS, MRS+1% arginine, and MRS+1% citrulline. Viable cells (CFU/mL), total cells (A_600_), pH level, and acidity of the culture were measured during 72 h (Figure 5). Cell viabilities of the three cultures were almost identical during 12~24 h, with a maximum cell viability of 8.99 log CFU/mL. However, cell viability of MRS dramatically decreased after 24 h, and 1.96 log CFU/mL was detected at 72 h. Viable cell counts among the others gradually decreased after 24 h; 6.60 log CFU/mL in MRS+1% arginine and 7.35 log CFU/mL in MRS+1% citrulline were detected at 72 h (Figure 5A).

Total cell growth levels of the three different cultures were significantly different (Figure 5B). Total cell growth of MRS+1% arginine (A_600_:3.70) was substantially higher than those of the other two cultures (A_600_:2.57 for MRS and A_600_:2.53 for MRS+1% citrulline). All three *W. koreensis* SK cultures reached stationary phase from 24 h, and the highest A_600_ values were obtained between 18~24 h.

The pHs (acidity %) of the cultures were pH 4.36 (1.05%) for MRS culture, pH 5.10 (0.65%) for MRS+1% citrulline culture, and pH 6.40 (0.31%) for MRS+1% arginine culture at 72 h (Figure 5C,D). Organic acid production due to cell growth in MRS resulted in a pH decrease. However, the pH of MRS+1% arginine culture slightly decreased until 12 h, after which it increased and reached its peak at 48 h, even though its total cell growth was significantly higher than those of the other two cultures (Figure 5).

Supplementation with arginine or citrulline has been shown to enhance bacterial growth, as bacteria use arginine and citrulline as energy, carbon, and nitrogen sources [40]. In this study, growth enhancement of *W. koreensis* SK was observed upon supplementation with arginine but not citrulline (Figure 5B). Arginine deiminase (ADI) converts L-arginine into L-citrulline and ammonia, after which L-citrulline is transferred to ornithine transcarbamoylase, which yields ornithine and carbamoyl phosphate. Carbamoyl phosphate is then converted into ammonia, carbon dioxide, and ATP by carbamate kinase [40]. Genes for the ADI pathway were identified in *W. koreensis* SK by RAST functional annotation (Figure 2B). Ammonia production by the ADI pathway of *W. koreensis* SK may have caused the increase in pH in MRS supplemented with arginine or citrulline. However, the pH of MRS culture decreased due to lack of ammonia production, which resulted in rapid reduction of cell viability at 72 h due to the weak acid tolerance of *W. koreensis* SK, as supported by Figure 4. The pH of *W. koreensis* SK culture in MRS supplemented with arginine (pH 6.40) at 72 h was clearly higher than those of the other two cultures (pH 4.36 for MRS, pH 5.10 for MRS+1% citrulline), and SK culture showed higher total cell growth (Figure 5). These results definitively show that arginine facilitates the growth of *W. koreensis* SK, whereas citrulline has no such effect even though citrulline supplementation caused a rise in pH along with ammonia production.

### 3.4. Arginine and Glucose Metabolism by W. koreensis SK

We determined the arginine catabolic products of *W. koreensis* SK in MRS, MRS+1% arginine, and MRS+1% citrulline cultures (Table 3). Added arginine and citrulline were converted into their metabolites and reached their highest amounts at 72 h. Most of the added arginine (MRS-A) was completely converted into citrulline (676.13 mg/L), ornithine (7174.73 mg/L), and ammonia (2321.34 mg/L) at 72 h. Added citrulline in MRS-C (6687.78 mg/L) gradually decreased in concentration (2155.59 mg/L) and was converted into ornithine (4254.92 mg/L) at 72 h. Glutamic acid levels of the three cultures gradually increased during 72 h of cultivation.

**Figure 6:**
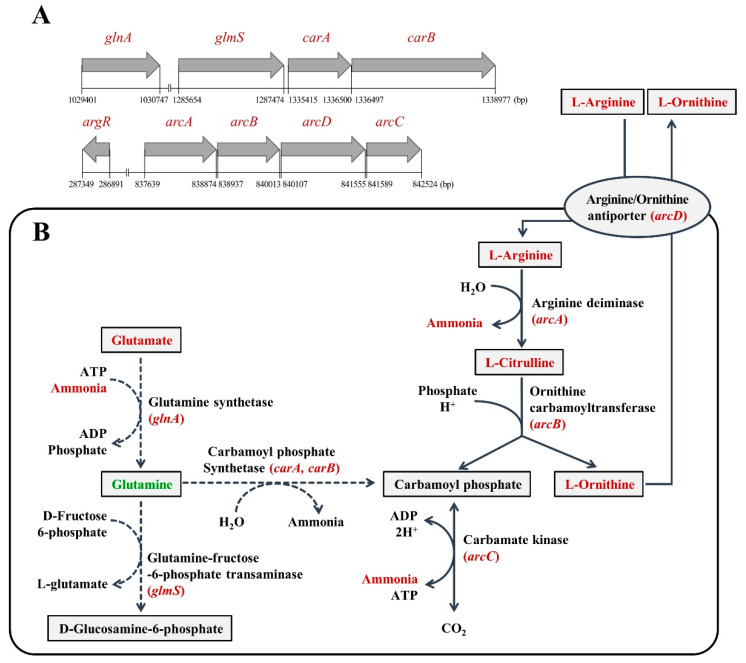
Schematic representation of ADI pathway-related gene cluster (**A**) and ADI pathway (**B**) in *W. koreensis* SK. Red character: identified compound or gene, green character: cannot identify, and black character: did not try to identify. Numbers in A indicate start and stop bases in the genome of *W. koreensis* SK. Solid arrow: proven pathway and dotted arrow: unproven pathway.

The RAST annotation results confirm arginine degradation via the ADI pathway, which is composed of ADI encoded by *arcA*, ornithine transcarbamoylase (encoded by *arcB*), and carbamate kinase (encoded by *arcC*), in *W. koreensis* SK. The ADI pathway of *W koreensis* SK was shown to be encoded by a cluster of four genes (*arcA-arcB-arcD-arcC*) and one arginine repressor gene (*argR)* located downstream of the cluster transcribed in the opposite direction (Figure 6A). It has been reported that clusters containing the structural genes encoding the ADI pathway are quite diverse, and the order of the genes differs among different species [41]. Analysis of the *arc* gene cluster found in the genomes of 1281 different bacterial species revealed the presence of 124 *arc* gene clusters and two types of L-arginine/L-ornithine exchangers, which belong to two different gene families (*arcD* and *arcE*) in bacteria. The majority of the *arc* gene clusters analyzed contain either *arcD* or *arcE*, but few (3/124) contain both [40]. Analysis of gene order in the 124 different bacterial clusters showed that the order of transcription was *arcA* followed by *arcB* and then *arc* with few exceptions. The *arcD* or *arcE* gene (and other *arc* genes) is added to this sequence or inserted in between. For example, the ADI gene cluster in *Lactobacillus brevis* contains *arcE1*-*arcA*-*arcB*-*arcD*-*arcT*-*arcC*-*argR* and *2CS*-*arcE2*-*arcD* in the opposite direction [42], whereas the ADI pathway of *Lactobcoccus lactis* is encoded by a cluster of nine genes, *arcD2-arcT-arcC2-arcC1-arcD1-arcB-arcA-argS* with *-argR* encoded in the opposite direction [40]. The order and direction of *arcA*, *arcB*, *arcC*, and *arcD* in *W. koreensis* SK were shown to be the same as those in other bacteria, but the numbers of these genes encoding the ADI pathway were found to be different. In particular, duplicated genes and other associated genes mostly of unknown function in other bacteria [40] were not found in *W. koreensis* SK.

Furthermore, carbamoyl phosphate synthetase (*carA and carB*), glutamine synthetase (*glnA*), and glutamine-fructose-6-phosphate transaminase (*glmS*) were detected in *W. koreensis* SK (Figure 6A). Glutamine can be synthesized from glutamate and ammonia by glutamate synthetase. However, glutamine was not detected in any of the three cultures at any time during cultivation (24 h and 72 h in Table 3 as well as in MRS+1% glutamate culture; data not shown) in the presence of glutamate, ammonia, and ATP. Based on the results, the arginine catabolic pathway of *W. koreensis* SK was constructed (with solid line) in Figure 6B based on genome analysis and determination of arginine catabolic products.

Glucose metabolites by *W. koreensis* SK were also determined (Table 3). Five compounds were detected; lactic acid and ethanol were significantly elevated during incubation, whereas the other compounds showed no significant changes. This result indicates that *W. koreensis* SK showed typical heterofermentative fermentation, in which glucose is converted into lactic acid, ethanol, and carbon dioxide. The highest amount of lactic acid was detected in MRS+1% arginine at 24 h and MRS at 72 h. While the highest amount of ethanol was detected in MRS+1% arginine, followed by MRS+1% citrulline, the lowest amount was detected in MRS culture at 24~72 h. The results of ethanol production by *W. koreensis* SK among the three cultures are consistent with those of total cell growth in Figure 5B. The improved cell growth caused high production of lactic acid and ethanol, followed by strong expression of the ADI pathway. This resulted in high production of ammonia, which was further neutralized by the lactic acid produced. As a consequence of neutralization with lactic acid and ammonia produced after 24 h, the lowest amount of lactic acid (7078.32 mg/L) was detected in MRS+1% arginine at 72 h. During arginine catabolism (Figure 6B), the concomitant production of ammonia by *W. koreensis* SK caused an increase in pH, which may promote survival of SK strain in an acidic environment. Based on the results, we can conclude that the detection of *W. koreensis* strains in the middle or late stage of kimchi fermentation (acidic condition) [23] could be attributed to their ammonia production ability, which neutralizes the produced acids according to cell growth, not to their acid tolerance ability.

L-Ornithine is an amino acid with a variety of significant functions, including stress reduction, improvement of sleep quality, physical fatigue reduction, wrinkle improvement, anti-obesity effect, and others [43]. Moreover, ornithine is a metabolic intermediate that plays a role in the urea cycle by detoxifying ammonia in order to reduce the level or ammonia in the bloodstream [43]. L-Citrulline has been reported to retard high glucose-induced endothelial senescence as well as provide nutritional support in malnourished patients, especially those who are aging and have sarcopenia [44]. In this study, ornithine production (7174.73 mg/L) by *W. koreensis* SK supplemented with 1% arginine was significantly higher than those (45~46 mg/L) of other *W. koreensis* strains [45]. Production of ornithine and citrulline by *W. koreensis* SK can be enhanced by optimization of this process through further experimentation. This unique property of *W. koreensis* SK implies that SK strain has potential as a functional starter culture or probiotic. The ADI pathway is widely utilized in bacteria, most abundantly in Lactobacillales [40]. We believe that this study is the first report to verify the ADI pathway of *W. koreensis* based on genome analysis and determination of arginine catabolic products.

## 4. Conclusions

*W. koreensis* strains were identified as dominant microorganisms in kimchi fermentation at low temperature (around 0 °C). The result indicates that *W. koreensis* strains play a major role in kimchi fermentation at low temperature. Regarding carbohydrate assimilation, *W. koreensis* strains showed a lesser degree (positive for 6~8 carbohydrates) of carbohydrate assimilation compared to *Lactobacillus* or *Leucnostoc* species. *W. koreensis* strains were found to utilize an alternative carbon source, the amino acid arginine, to obtain energy. In particular, *W. koreensis* SK selected among the 50 isolates in this study showed advanced arginine utilization. Accordingly, arginine supplementation improved bacterial growth and resulted in high production of ornithine. *W. koreensis* SK was able to grow well under mesophilic as well as psychrophilic conditions. Furthermore, *W. koreensis* SK was found to be safe for human consumption and beneficial due to its functional material production (ornithine) ability. The results of this study indicate that *W. koreensis* SK may be a promising starter culture for fermented vegetables or fruits at low temperature as well as a probiotic candidate.

## Figures and Tables

**Table 1:** Comparison of general genome features of *W. koreensis* SK and other *W. koreensis* strains.

Feature	SK		KACC 15510		WiKim0080		CBA3615	
	Chromo- Some	Plasmid	Chromo- Some	Plasmid	Chromo- Some	Plasmid	Chromo- Some	Plasmid
Genome size (bp)	1,451,607	10,481	1,422,478	18,992	1,495,237	31,842 9934	1,491,842	19,971
G+C content (%)	35.5	36.9	35.5	36.7	35.5	40.0 27.5	35.4	38.7
Total genes (no.)	1389	11	1379	24	1454	32 11	1443	27
Protein coding sequences (no.)	1318	11	1308	24	1382	32 11	1371	27
rRNAs (no.)	15	-	15	-	15	-	15	-
tRNAs (no.)	56	-	56	-	57	-	57	-
GenBank Accession No.	CP043431	CP043432	CP002899	CP002900	CP026847	CP026848 CP026849	CP046070	CP046071
Assembly level ^1^	Complete		Complete		Complete		Chromosome	
Source	Kimchi		Kimchi		Kimchi		Kimchi	
Reference	This study ^2^		[27]		[10]		[28]	

^1^ Complete; all chromosomes are gapless and have no runs of 10 or more ambiguous bases, there are no unplaced or unlocalized scaffolds, and all the expected chromosomes are present. Chromosome; there is sequence for one or more chromosomes. This could be a completely sequenced chromosome without gaps or a chromosome containing scaffolds or contigs with gaps between them. There may also be unplaced or unlocalized scaffolds. ^2^ *W. koreensis* was deposited in The Korean Collection for Type Cultures (Jeongeup, Korea) under the accession No. KCTC14235BP.

**Table 2:** ORFs and their deduced proteins on plasmid pSK from *W. koreensis* SK.

ORF	ORF Length (a.a)	Related Protein (Length of a.a)	Identity (%)	Accession No. of Related Protein
1	411	mobilization protein [*Weissella koreensis*] (411)	99.76	WP_104914736.1
2	384	MULTISPECIES: RepB family plasmid replication initiator protein [Leuconostocaceae] (384)	98.18	WP_102753750.1
3	151	CopY/TcrY family copper transport repressor [*Weissella confusa*] (151)	100.00	WP_135469337.1
4	58	heavy-metal-associated domain-containing protein [*Weissella soli*] (58)	100.00	WP_147153977.1
5	692	MULTISPECIES: cadmium-translocating P-type ATPase [Leuconostocaceae] (692)	99.86	WP_004909401.1
6	127	MULTISPECIES: glyoxalase/bleomycin resistance/extradiol dioxygenase family protein [*Weissella*] (127)	100.00	WP_104914740.1
7	242	MULTISPECIES: fructose permease [*Leuconostoc*] (242)	98.76	WP_071952482.1

Functional annotation of coding sequences (CDSs) on plasmid pSK was performed using basic local alignment search tool (BlastX).

**Table 3:** ADI and glucose metabolites produced by *W. koreensis* SK. Unit: mg/L

Substrate	Culture	Content	Incubation Time		
			0 h	24 h	72 h
Arginine /Citrulline	MRS	Glutamic acid	614.69 ± 17.96 ^a,C^	666.74 ± 9.16 ^a,B^	719.00 ± 16.48 ^a,A^
		Glutamine	N.D ^1^	N.D	N.D
		Citrulline	11.38 ± 3.85 ^c,A^	3.07 ± 0.85 ^c,B^	3.91 ± 0.20 ^c,B^
		Arginine	301.38 ± 62.79 ^b,A^	7.48 ± 2.65 ^b,B^	5.09 ± 1.74 ^b,B^
		Ornithine	38.78 ± 10.09 ^b,B^	93.25 ± 13.10 ^c,A^	99.16 ± 8.37 ^c,A^
		NH_3_	453.06 ± 49.30 ^a,A^	462.52 ± 46.27 ^b,A^	443.97 ± 10.23 ^c,A^
	MRS-A	Glutamic acid	611.92 ± 17.25 ^a,C^	677.89 ± 5.39 ^a,B^	739.63 ± 11.00 ^a,A^
		Glutamine	N.D	N.D	N.D
		Citrulline	47.90 ± 11.75 ^b,C^	841.70 ± 75.64 ^b,A^	676.13 ± 12.60 ^b,B^
		Arginine	7713.34 ± 34.57 ^a,A^	62.25 ± 9.10 ^a,B^	41.68 ± 1.07 ^a,B^
		Ornithine	55.81 ± 21.42 ^b,B^	6569.52 ± 327.28 ^a,A^	7174.73 ± 148.56 ^a,A^
		NH_3_	465.11 ± 8.64 ^a,B^	2334.97 ± 182.86 ^a,A^	2321.34 ± 143.57 ^a,A^
	MRS-C	Glutamic acid	617.07 ± 16.83 ^a,C^	676.51 ± 12.01 ^a,B^	725.21 ± 10.52 ^a,A^
		Glutamine	N.D	N.D	N.D
		Citrulline	6687.78 ± 5.11 ^a,A^	4504.69 ± 199.02 ^a,B^	2155.59 ± 69.07 ^a,C^
		Arginine	229.10 ± 0.08 ^b,A^	5.01 ± 1.60 ^b,B^	4.95 ± 0.24 ^b,B^
		Ornithine	1010.71 ± 63.32 ^a,A^	3048.81 ± 76.68 ^b,B^	4254.92 ± 193.10 ^b,C^
		NH_3_	479.51 ± 32.06 ^a,B^	697.32 ± 112.75 ^b,B^	938.11 ± 29.22 ^b,A^
Glucose	MRS	Lactic acid	209.00 ± 9.85 ^a,C^	5917.63 ± 201.77 ^b,B^	7556.81 ± 31.06 ^a,A^
		Formic acid	144.36 ± 1.12 ^a,B^	180.37 ± 1.05 ^b,A^	198.10 ± 17.70 ^a,A^
		Acetic acid	4396.08 ± 49.64 ^a,C^	4538.84 ± 4.50 ^a,B^	4730.90 ± 28.87 ^a,A^
		EtOH	N.D	354.56 ± 10.03 ^b,B^	431.56 ± 1.16 ^b,A^
		Citric acid	1947.97 ± 38.88 ^a,A^	1870.40 ± 22.09 ^a,A^	1895.47 ± 38.77 ^a,A^
	MRS-A	Lactic acid	210.14 ± 2.50 ^a,B^	6965.00 ± 255.56 ^a,A^	7078.32 ± 9.72 ^b,A^
		Formic acid	145.69 ± 19.66 ^a,B^	233.16 ± 4.01 ^a,A^	228.98 ± 15.42 ^a,A^
		Acetic acid	4351.16 ± 54.43 ^a,B^	4539.66 ± 25.54 ^a,A^	4534.56 ± 34.99 ^bA^
		EtOH	N.D	458.74 ± 23.58 ^a,A^	464.78 ± 4.60 ^a,A^
		Citric acid	1932.38 ± 57.61 ^a,B^	1817.38 ± 7.87 ^a,A,B^	1832.65 ± 13.49 ^a,b,A^
	MRS-C	Lactic acid	194.64 ± 3.72 ^a,C^	5339.90 ± 192.18 ^b,B^	7166.88 ± 223.21 ^a,b,A^
		Formic acid	148.02 ± 30.62 ^a,A^	207.75 ± 14.47 ^a,b,A^	197.35 ± 3.49 ^a,A^
		Acetic acid	4220.48 ± 238.05 ^a,A^	4475.87 ± 2.93 ^b,A^	4481.72 ± 69.85 ^b,A^
		EtOH	N.D	323.44 ± 15.38 ^b,B^	444.94 ± 5.91 ^b,A^
		Citric acid	1825.29 ± 108.46 ^a,A^	1823.65 ± 35.44 ^a,A^	1767.00 ± 41.11 ^a,A^

*W. koreensis* SK was cultivated in MRS, MRS+1% arginine (MRS-A), and MRS+1% citrulline (MRS-C) at 30 °C for 72 h. Each content from the culture supernatant was measured at 0, 24, and 72 h as described in Materials and Methods. Values are mean ± SD from duplicate determination. Means with different letters are significantly different (*p* < 0.05) by one-way ANOVA, followed by Duncan’s multiple range test; a~c: means for culture media (row); A–C: means for incubation time (column). ^1^ N.D: Not detected.

## Supplementary Materials

The following are available online at https://www.mdpi.com/2076-2607/8/8/1147/s1, Figure S1: HPLC analysis of biogenic amine from *W. koreensis*, Table S1: Isolated LAB strains and their 16s rRNA gene sequences similarities with sequences in the GenBank database, Table S2: Carbohydrate assimilation of LAB.
