# Supplementary material for: Characterization of Weissella koreensis SK Isolated from Kimchi Fermented at Low Temperature (around 0 °C) Based on Complete Genome Sequence and Corresponding Phenotype
Source: Microorganisms. 2020 Jul 29;8(8):1147. doi: 10.3390/microorganisms8081147 (PMC7464874; doi:10.3390/microorganisms8081147)
Supplement: Supplementary file 1 [file microorganisms-08-01147-s001.pdf]

Supplementary data

Figure S1. HPLC analysis of biogenic amine from *W. koreensis*. (A) Biogenic amine standards, PUT; putrescine, HIS; histamine, AGM; agmatine, and IS; 1,7-diaminoheptane as an internal standard. *W. koreensis* SK was incubated in (B) MRS+1% arginine, (C) MRS+1% ornithine, and (D) MRS+1% histidine at 30°C for 48 h, after which biogenic amine levels in the culture broths were determined.

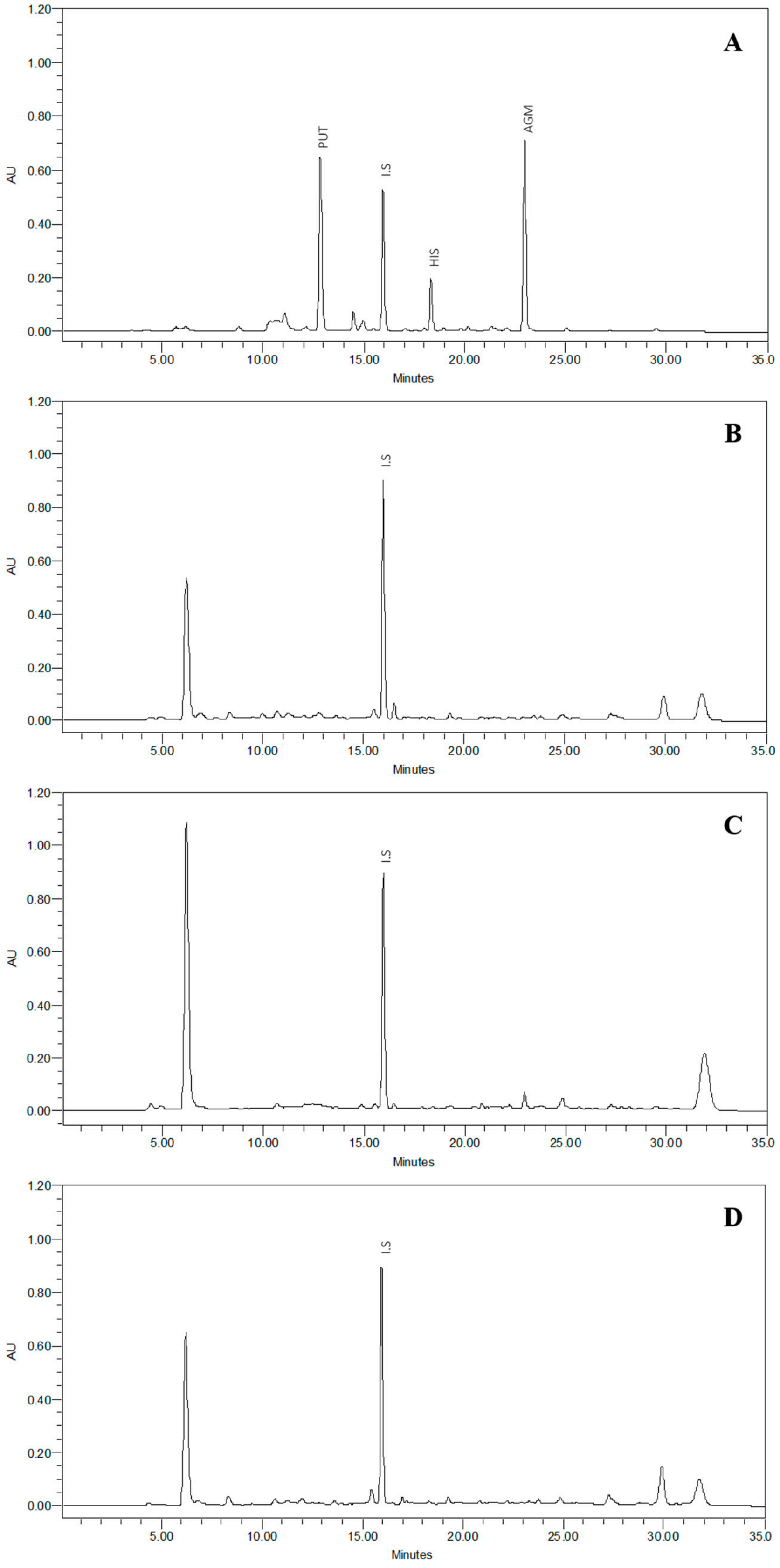
A
B
C
D
PUT
I.S
HIS
AGM
AU
Minutes
0.00
0.20
0.40
0.60
0.80
1.00
1.20
5.00
10.00
15.00
20.00
25.00
30.00
35.0

Figure S1.

Table S1. Isolated LAB strains and their 16s rRNA gene sequences similarities with sequences in the GenBank database.

Sampling location		Kimchi sample	Isolate	Length of 16s rRNA gene (bp)	Similarity* (%)	E value
Province	City/District					
Seoul/ Incheon/ Gyeonggi	Seoul	Kimchi 1	LAB 1	1485	100	0.0
	Seoul	Kimchi 2	LAB 2	1269	100	0.0
	Incheon	Kimchi 3	LAB 3	1268	100	0.0
	Ganghwa	Kimchi 4	LAB 4	1504	100	0.0
	Bucheon	Kimchi 5	LAB 5	1442	100	0.0
			LAB 6	1514	100	0.0
	Siheung	Kimchi 6	LAB 7	1492	100	0.0
	Anseong	Kimchi 7	LAB 8	1490	100	0.0
	Yangpyeong	Kimchi 8	LAB 9	1379	100	0.0
	Pocheon	Kimchi 9	LAB 10	1489	100	0.0
Gangwon	Goseong	Kimchi 10	LAB 11	1501	100	0.0
	Sokcho	Kimchi 11	LAB 12	1494	100	0.0
	Yanggu	Kimchi 12	LAB 13	1480	100	0.0
	Wonju	Kimchi 13	LAB 14	1497	100	0.0
	Taebaek	Kimchi 14	LAB 15	1288	100	0.0
	Taebaek	Kimchi 15	LAB 16	1307	100	0.0
	Pyeongchang	Kimchi 16	LAB 17	1309	100	0.0
	Hongcheon	Kimchi 17	LAB 18	1460	100	0.0
			LAB 19	1270	100	0.0
	Gangneung	Kimchi 18	LAB 20	1511	100	0.0
Chungcheong	Nonsan	Kimchi 19	LAB 21	1356	100	0.0
		Kimchi 20	LAB 22	1360	100	0.0
	Seosan	Kimchi 21	LAB 23	1518	100	0.0
	Taean	Kimchi 22	LAB 24	1453	100	0.0
			LAB 25	1385	100	0.0
	Asan	Kimchi 23	LAB 26	1521	100	0.0
	Jecheon	Kimchi 24	LAB 27	1376	100	0.0
	Chengyang	Kimchi 25	LAB 28	1378	100	0.0
			LAB 29	1362	100	0.0
	Cheongju	Kimchi 26	LAB 30	1523	100	0.0
	Cheongju	Kimchi 27	LAB 31	1517	100	0.0
Daegu/ Busan/ Gyeongsang	Daegu	Kimchi 28	LAB 32	1383	100	0.0
	Daegu	Kimchi 29	LAB 33	1278	100	0.0
	Busan	Kimchi 30	LAB 34	1378	100	0.0
	Gimhae	Kimchi 31	LAB 35	1373	100	0.0
	Andong	Kimchi 32	LAB 36	1369	100	0.0
	Yecheon	Kimchi 33	LAB 37	1518	100	0.0
	Pohang	Kimchi 34	LAB 38	1288	100	0.0
Gwangju/ Jeolla	Gwangju	Kimchi 35	LAB 39	1299	100	0.0
	Gwangju	Kimchi 36	LAB 40	1373	100	0.0
	Gwangju	Kimchi 37	LAB 41	1503	100	0.0
	Gokseong	Kimchi 38	LAB 42	1517	100	0.0
	Naju	Kimchi 39	LAB 43	1512	100	0.0
	Jangseong	Kimchi 40	LAB 44	1386	100	0.0
	Haenam	Kimchi 41	LAB 45	1516	100	0.0
			LAB 46	1356	100	0.0
	Hwasun	Kimchi 42	LAB 47	1513	100	0.0
	Iksan	Kimchi 43	LAB 48	1499	100	0.0
	Imsil	Kimchi 44	LAB 49	1515	100	0.0
	Jeonju	Kimchi 45	LAB 50	1386	100	0.0

*16s rRNA sequences of isolates were compared with those of *W. koreensis* JCM 11263^T.

Table S2. Carbohydrate assimilation of LAB.

NO.	LAB strain / Carbohydrates	*W. koreensis*															
		KACC 11853^T	LAB1	LAB 3	LAB 6	LAB11	LAB14	LAB18	LAB 23	LAB26	LAB31	LAB34	LAB37	LAB41	LAB43	LAB45	LAB48
1	Glycerol	-	-	-	-	-	-	-	-	-	-	-	-	-	-	-	-
2	Erythritol	-	-	-	-	-	-	-	-	-	-	-	-	-	-	-	-
3	D-Arabinose	-	-	-	-	-	-	-	-	-	-	-	-	-	-	-	-
4	L-Arabinose	+	+	-	+	+	+	+	+	+	+	+	+	+	+	+	+
5	Ribose	+	+	+	+	+	+	+	+	+	+	+	+	+	+	+	+
6	D-Xylose	+	+	+	+	+	+	+	-	+	+	+	+	+	+	+	+
7	L-Xylose	-	-	-	-	-	-	-	-	-	-	-	-	-	-	-	-
8	Adonitol	-	-	-	-	-	-	-	-	-	-	-	-	-	-	-	-
9	Methyl-BD-xylopyranosicle	-	-	-	-	-	-	-	-	-	-	-	-	-	-	-	-
10	D-Galactose	-	-	-	-	-	-	-	-	-	-	-	-	-	-	-	-
11	D-Glucose	+	+	+	+	+	+	+	+	+	+	+	+	+	+	+	+
12	D-Fructose	+	+	-	-	+	+	+	-	+	+	+	+	+	+	+	+
13	D-Mannose	+	+	+	+	+	+	+	+	+	+	+	+	+	+	+	+
14	L-Sorbose	-	-	-	-	-	-	-	-	-	-	-	-	-	-	-	-
15	Rhamnose	-	-	-	-	-	-	-	-	-	-	-	-	-	-	-	-
16	Dulcitol	-	-	-	-	-	-	-	-	-	-	-	-	-	-	-	-
17	Inositol	-	-	-	-	-	-	-	-	-	-	-	-	-	-	-	-
18	Mannitol	-	-	-	-	-	-	-	-	-	-	-	-	-	-	-	-
19	Sorbitol	-	-	-	-	-	-	-	-	-	-	-	-	-	-	-	-
20	α-Methyl-D-mannoside	-	-	-	-	-	-	-	-	-	-	-	-	-	-	-	-
21	α-Methyl-D-Glucoside	-	-	-	-	-	-	-	-	-	-	-	-	-	-	-	-
22	N-Acetyl glucosamine	+	+	+	+	+	+	+	+	+	+	+	+	+	+	+	+
23	Amygdaline	-	-	-	-	-	-	-	-	-	-	-	-	-	-	-	-
24	Arbutin	-	-	-	-	-	-	-	-	-	-	-	-	-	-	-	-
25	Esculine	-	-	-	-	-	-	-	-	-	-	-	-	-	-	-	-
26	Salicine	-	-	-	-	-	-	-	-	-	-	-	-	-	-	-	-
27	Cellobiose	-	-	-	-	-	-	-	-	-	-	-	-	-	-	-	-
28	Malotse	-	-	-	-	-	-	-	-	-	-	-	-	-	-	-	-
29	Lactose	-	-	-	-	-	-	-	-	-	-	-	-	-	-	-	-
30	Melibiose	-	-	-	-	-	-	-	-	-	-	-	-	-	-	-	-
31	Sucrose	-	-	-	-	-	-	-	-	-	-	-	-	-	-	-	-
32	Trehalose	-	-	-	-	-	-	-	-	-	-	-	-	-	-	-	-
33	Inuline	-	-	-	-	-	-	-	-	-	-	-	-	-	-	-	-
34	Melezitose	-	-	-	-	-	-	-	-	-	-	-	-	-	-	-	-
35	Rafiinose	-	-	-	-	-	-	-	-	-	-	-	-	-	-	-	-
36	Starch	-	-	-	-	-	-	-	-	-	-	-	-	-	-	-	-
37	Glycogen	-	-	-	-	-	-	-	-	-	-	-	-	-	-	-	-
38	Xylitol	-	-	-	-	-	-	-	-	-	-	-	-	-	-	-	-
39	β-Gentiobiose	-	-	-	-	-	-	-	-	-	-	-	-	-	-	-	-
40	D-Turanose	-	-	-	-	-	-	-	-	-	-	-	-	-	-	-	-
41	D-Lyxose	-	-	-	-	-	-	-	-	-	-	-	-	-	-	-	-
42	D-Tagatose	-	-	-	-	-	-	-	-	-	-	-	-	-	-	-	-
43	D-Fucose	-	-	-	-	-	-	-	-	-	-	-	-	-	-	-	-
44	L-Fucose	-	-	-	-	-	-	-	-	-	-	-	-	-	-	-	-
45	D-Arabitol	-	-	-	-	-	-	-	-	-	-	-	-	-	-	-	-
46	L-Arabitol	-	-	-	-	-	-	-	-	-	-	-	-	-	-	-	-
47	Gluconate	w	w	w	w	w	w	w	w	w	w	w	w	w	w	w	w
48	2 Keto-gluconate	-	-	-	-	-	-	-	-	-	-	-	-	-	-	-	-
49	5 keto-gluconate	-	-	-	-	-	-	-	-	-	-	-	-	-	-	-	-

Carbohydrate assimilation of LAB was determined using API 50 CHL system; w, weakly positive; +, positive; -, negative.
